# “No Time for Myself”: Personality Moderates Associations Between Positive Solitude and Parental Well-being

**DOI:** 10.21203/rs.3.rs-7200119/v1

**Published:** 2025-07-31

**Authors:** Theresa Pauly

**Affiliations:** 1 Department of Gerontology, Simon Fraser University

## Abstract

This study aimed to examine whether daily experiences of positive solitude—defined as time to oneself—relate to lower negative affect and healthier cortisol patterns in parents of underage children, and whether personality traits moderate these associations. A sample of 318 parents (Mage = 40.06 years; 45% male) with underage children completed up to 8 consecutive days of daily diaries (mood, positive solitude, stress exposure) and up to 4 days of saliva sampling (4 times/day) for cortisol analysis. Multilevel modeling examined within-person links between positive solitude, negative affect, and cortisol wake-evening slopes, controlling for daily stress. Results showed that on days when they had time to themselves, parents experienced lower negative affect and steeper cortisol slopes, indicating better stress recovery. The reduction in negative affect with positive solitude was stronger for parents high in neuroticism and openness, and high neuroticism was also linked with a stronger association between solitude and cortisol slopes. Findings underscore the potential restorative value of daily positive solitude for parents, particularly those high in neuroticism and openness. In the context of the high demands of parenting, positive solitude may serve as a valuable resource for emotional renewal, self-care, self-connection, and recovery from daily parenting stress.

There is a common belief that spending time alone is inherently lonely, and that those who prefer solitude should be pitied. U.S. newspaper headlines are ten times more likely to portray being alone in a negative rather than a positive light^1^. However, many people actively choose solitude over social interaction. Solitude is a complex experience that can encompass a wide range of emotional states. Philosophers, artists, and spiritual practitioners have long recognized its dual nature—both enriching and challenging^2,3^. It is important to distinguish between related concepts: aloneness refers to the physical absence of others, while solitude describes the lack of social contact or interaction, which can occur even when others are physically present^4–6^. Solitude differs from loneliness, which is the subjective feeling of lacking meaningful social connection^7^.

Solitude has been shown to support emotional recovery, relaxation, agency, creativity, concentration on cognitively demanding tasks, and self-reflection^8,9^, and it may be especially beneficial for parents who face constant demands on their time and attention. This study explores the role of positive solitude—defined as time to oneself—for the daily emotional and physiological well-being of parents of underage children, and examines whether these associations are moderated by personality.

## Benefits of Positive Solitude

Early research on solitude emphasized privacy as a way individuals regulate social contact. Pedersen^10^ identified solitude as one of six primary types of privacy and delineated five core functions: autonomy, confiding, rejuvenation, contemplation, and creativity. The Social Affiliation Model^11^ offers a homeostatic view of solitude and social interaction. Much like hunger drives food intake, humans are thought to monitor and regulate social engagement, seeking to maintain an optimal range. When this balance is disrupted—either by too much solitude or too much social contact—people are motivated to reestablish equilibrium. Similarly, the Communicate Bond Belong Theory^12^ suggests that social engagement is governed by both the desire to belong and the need to conserve energy. Individuals are thought to have a limited amount of social energy, and when it is depleted, they are more likely to seek solitude. In support of this view, Luo et al.^9^ found that, in a sample of 118 older adults, longer-than-usual social interactions were both preceded and followed by longer-than-usual solitude episodes, suggesting a natural rhythm of alternating between social engagement and alone time.

More recent research has expanded our understanding of the positive functions of solitude beyond mere privacy regulation. Weinstein et al.^13^ analyzed solitude experiences in over 2,000 individuals aged 13 to 85, identifying several domains in which solitude contributes to well-being: (a) Competence: Solitude fosters skill development, self-efficacy, and the pursuit of meaningful leisure activities; (b) Autonomy: Time alone provides a sense of choice, freedom from social pressure, and relief from external responsibilities, enabling deeper self-connection; (c) Self-growth: Solitude supports self-reflection, the development of coping strategies, and spiritual or existential exploration; (d) Self-care: Many use solitude to recharge and attend to their physical, mental, and emotional needs. Experience sampling studies that track people’s emotions throughout their daily lives—both during solitude and while with others—have found that individuals tend to report greater levels of low-arousal positive emotions, such as calmness, contentment, and relaxation, when they are alone^14,15^.

Positive experiences during solitary moments are referred to as *positive solitude*. Palgi et al.^16^ describe positive solitude as situations where individuals intentionally choose to spend time alone, such as reading, going for a walk, or working on their computer, or find ways to enrich their solitude by engaging in personally meaningful or enjoyable activities like listening to music. Similarly, Ost Mor et al.^17^ conceptualize positive solitude as “the choice to dedicate time to a meaningful, enjoyable activity or experience conducted by oneself” (p. 15), emphasizing that such experiences may be spiritual, recreational, or functional in nature, and can occur regardless of the physical presence of others. Individuals who report higher positive solitude, report lower levels of depressive symptoms^18^, higher levels of mindfulness^19^ and flourishing^20^, and better health and well-being^16^.

Despite these benefits, there is substantial evidence that excessive solitude can have detrimental effects. Perhaps the most widely acknowledged risk is loneliness—the subjective feeling that one’s desired level of social connection is unmet^7^. Loneliness has been linked to a range of negative outcomes, including increased risk for mental health disorders, cognitive decline, and cardiovascular disease^21,22^.

On the other hand, insufficient solitude also poses challenges. This concept has been termed aloneliness, and is defined as “the negative feelings that may arise from the perception that one is not getting to spend *enough* time alone”^23^, p. 17. Without enough time alone, individuals may struggle to decompress, reflect, or disengage from daily demands. Accordingly, aloneliness has been associated with increased levels of negative affect, stress, and depressive symptoms^23^ and lower levels of life and family satisfaction^24^. One stage in life during which positive solitude might be particularly scarce is when parenting young children.

## Benefits of Positive Solitude for Parenting

The transition to parenthood—especially during a child’s first year—results in profound shifts in daily routines, personal identity, and time use^25^. New parents consistently report an overwhelming reorganization of their lives as caregiving responsibilities become all-consuming^25,26^. Daily care for young children often demands near-constant attention, leaving little time for personal needs, rest, or recuperation^26^. These changes are not only logistical but deeply emotional and psychological; parents often struggle to recalibrate their roles and routines, and to adjust to the loss of their former life and activities^25^. Thus, not surprisingly, parental stress is robustly linked to reduced well-being^27^.

One of the most pronounced consequences of the parenthood transition is the reduction in leisure activities and personal time. Multiple studies show that both mothers and fathers experience a substantial decline in recreational and restorative activities^24,28–30^. However, mothers tend to bear a disproportionate burden of this shift. Compared to fathers, they are twice as likely to report that they do not have enough time for themselves^24^, exercise less frequently^30^, and report more difficulty mentally disengaging from caregiving demands and other household responsibilities even when physically alone^31,32^.

Consequently, having enough time to oneself might have important implications for well-being in parents. Parental stress often involves feelings of being overwhelmed, anxious, and depleted—emotions that positive solitude can help down-regulate^14,32^. Positive solitude also allows for energy restoration and mental reset^12,14^. Even brief solitary activities—such as napping, resting, or listening to calming music—might provide relief from daily demands. These moments of personal space might help replenish depleted resources and restore the energy required for continued caregiving^32^.

Previous research has primarily examined the links between solitude or leisure activities with parents’ health and well-being using either cross-sectional methods or long-term longitudinal designs that span multiple years e.g.,^24,30^. However, these approaches fall short in capturing the dynamic, short-term, and context-dependent nature of positive solitude—especially its potential function as a source of restoration and emotional regulation.

The current study addresses this limitation by employing an experience sampling design, which offers a more fine-grained, real-time window into how variations in daily positive solitude relate to fluctuations in psychological and physiological well-being. By tracking participants’ experiences across multiple days, this method captures within-person variability and reveals how positive solitude may function as a coping resource on a day-to-day basis, rather than assuming stable, trait-like associations^33^.

Furthermore, prior research has rarely investigated *how* time to oneself might protect from long-term ramifications of parenting stress on physical health. The present study begins to fill this gap by examining daily cortisol patterns—a key biomarker of stress and recovery^34^. Cortisol marks activity of the hypothalamic-pituitary-adrenal (HPA) axis and follows a diurnal rhythm, peaking shortly after waking and declining steadily throughout the day^35^. On days with acute stress or heightened negative emotions, this rhythm flattens, signaling reduced physiological recovery^36,37^. Overall, flatter diurnal cortisol slopes have been linked to a wide range of adverse mental and physical health outcomes, including increased risk for depression, impaired immune functioning, obesity, cancer, and cardiovascular disease^38^. While leisure activities such as music listening have been shown to reduce cortisol in everyday life^39^, the potential stress-buffering effects of positive solitude during daily life remain understudied. This study examined whether positive solitude is linked with lower daily negative affect and steeper cortisol slopes in parents of underage children. However, the nature of these associations may vary from one parent to another.

## Personality Differences in Benefits of Positive Solitude

Personality might play a role in shaping responses to the stresses of parenthood. Longitudinal and cross-sectional studies alike show that individuals high in neuroticism—characterized by heightened emotional instability, vulnerability to stress, and anxiety—are more likely to experience elevated parental stress and burnout^40–42^. Conversely, extraversion—characterized by energy, positive emotions, sociability, and the tendency to seek stimulation and the company of others—is generally protective with extraverted parents report lower levels of parenting stress^42^. Conscientiousness—characterized by self-discipline, carefulness, thoroughness, organization, and a desire to achieve goals—has been related both reduced risk^41^ as well as increased risk (particularly for the trait of meticulousness^40^) of parental burnout.

Individual differences might thus also play a role in who seeks and benefits from positive solitude when faced with parental stress. Traits such as sensory processing sensitivity and affinity for aloneness have been linked to a greater tendency to seek solitude and benefit from it emotionally^43,44^. Among the Big Five personality traits, introversion—characterized by lower sociability and assertiveness, and greater tendencies toward reserved, reflective, and solitary behavior—would intuitively seem most closely linked to the experience of positive solitude^45^. However, several studies have found no significant relationship between introversion and enjoyment of solitude, self-determined motivation for solitude, or preference for solitude^43,44,46^. More recent research with a representative U.S. sample (N = 501) has shown that personality traits might relate differentially to solitude functions^47^, with extraversion and neuroticism showing the strongest associations. Specifically, the authors found that individuals high in neuroticism viewed solitude as important for emotion regulation and escape, while those high in extraversion placed less value on solitude for relaxation or avoiding unpleasant interactions. Openness, on the other hand, was linked to using solitude for creativity and self-discovery^47^. Consequently, another aim of this study is to examine whether daily associations between positive solitude and negative affect as well as cortisol slopes are moderated by personality traits.

## The Current Study

The present study investigates how positive solitude—defined as time to oneself—relates to daily emotional and physiological well-being of parents of underage children. Drawing on experience sampling data from a subsample (N = 318) of the Midlife Development in the United States (MIDUS) Study, collected between 2011 and 2014, participants reported their daily experiences across eight consecutive days, with salivary cortisol samples collected across 4 days. Guided by recent work highlighting the role of individual differences in the function and experience of solitude^47^, the study further examines how the associations between solitude and daily well-being may be moderated by personality.

It was hypothesized that (H1) on days when individuals experience positive solitude, they would report lower negative affect and exhibit steeper diurnal cortisol slopes compared to days without positive solitude. Furthermore, (H2) these associations would vary depending on personality traits.

## Methods

### Procedures and Participants

Data for the current study are from the Midlife Development in the United States Survey (MIDUS; http://midus.wisc.edu), which began in 1994 and has collected data from the same participants across three waves since (MIDUS1: 1994–1995, N = 7,108 adults aged 25–74; MIDUS2: 2004–2006, N = 5,555; MIDUS3: 2013–2015, N = 3,683). Between 2011 and 2014, an additional sample (Refresher Cohort) of 3,577 adults aged 25 to 74 was recruited to replenish the number of middle-aged adults in the original MIDUS cohort. Participants were recruited through random dial digits. The MIDUS Refresher survey used the same assessments as the original study, where participants first completed 30 min baseline phone interviews followed by self-administered questionnaires via mail. A subsample of participants (N = 782) took part in a Daily Diary project for which participants completed phone surveys on 8 consecutive evenings. These daily surveys covered various aspects of their daily lives, including stressors, emotions, physical symptoms, and social interactions. MIDUS data collection is reviewed and approved by the Education and Social/Behavioral Sciences and the Health Sciences IRBs at the University of Wisconsin-Madison. Secondary data analysis for this study was approved by the Research Ethics Board at Simon Fraser University.

The current study uses data from the Refresher Daily Diary subproject only, as daily time to oneself was not measured in any of the three other MIDUS surveys. The analytical sample included individuals that have at least one underage child living in their household (biological child, adopted child, step child, or child of partner). One person was missing information on personality, resulting in a sample size of n = 2,299 surveys from N = 318 participants. Participants had an average age of 40.06 years (*SD* = 7.54), 45% were male, the average annual household income was $97,435.71 (*SD* = $65,183.49), and 81% identified as White. They lived with an average of 2 underage children (*M* = 2.02, *SD* = 1.15) and the youngest child was 7.61 years old, on average (*SD* = 5.19). This sample of parents was significantly younger, more likely to be White, had higher average household income, better self-rated health, less time to themselves, lower average negative affect, and steeper cortisol slopes than the remainder of the Refresher Daily Diary sample. Samples did not differ in sex. Participants completed 7.23 out of 8 scheduled surveys on average (*SD* = 1.66).

### Measures

#### Positive Solitude

As part of the 8 daily surveys, participants were asked: “Since this time yesterday, did you have the opportunity to take time for yourself?”. Answer options were “Yes” (1) or “No” (0).

#### Daily Negative Affect

Participants were also asked to report their mood using 14 items related to negative affect, with the prompt: “How much of the time today did you feel …?” Responses were rated on a scale from 0 (not at all) to 4 (all the time). The items included: restless or fidgety, nervous, worthless, so sad nothing could cheer you up, everything was an effort, hopeless, lonely, afraid, jittery, irritable, ashamed, upset, angry, and frustrated. Items were drawn from the Nonspecific Psychological Distress Scale^48^ and a modified version of the Positive and Negative Affect Schedule^49^. Daily negative affect was calculated by averaging the scores across all items, with higher scores indicating higher levels of negative affect (possible range: 0–4). Reliability indices of repeated measures as described by Cranford et al.^50^ were high at both the between-person (R_KF_ = .86) and within-person level (R_C_ = .84).

#### Wake-Evening Cortisol Slope

Participants collected four saliva samples on days two through five of their 8-day daily life assessments at the following times: immediately upon waking, 30 minutes post-waking, before lunch, and before bedtime. Collection times were logged and verified through daily diary interviews. Saliva samples were analyzed for cortisol with a commercially available luminescence immunoassay (IBL, Hamburg, Germany) at the Biological Psychology Laboratory at the Technical University of Dresden. Inter-assay and intra-assay coefficients of variance were below 5%. For more details about cortisol collection and processing see Almeida et al.^51^ and Dmitrieva et al.^52^. Cortisol data were excluded if participants demonstrated non-compliance or out-of-range cortisol values. In line with previous MIDUS studies^52^, any sample with cortisol ≥60 nmol/L was treated as missing, and days were excluded if participants awoke before 4am or after 11am, were awake for less than 12 hours or more than 20 hours, if the first waking sample was delayed by more than 15 minutes after waking, or if cortisol levels increased by more than 10 nmol/L at bedtime compared to post-waking levels. The final analytic sample consisted of 788 valid cortisol days, completed by 255 participants. On average, participants provided 3.08 (SD = 1.02) days of cortisol data. The wake-evening slope for each day was calculated by subtracting the morning from the evening cortisol value, dividing by time elapsed between the two samples^53^.

#### Personality

Participants rated how well 26 self-descriptive adjectives described them on a scale from 1 (“a lot”) to 4 (“not at all”). These adjectives assessed the Big 5 personality traits: neuroticism (4 items), extraversion (5 items), openness to experience (7 items), conscientiousness (5 items), and agreeableness (5 items). For information on scale construction see Lachman & Weaver^54^. Personality trait scores were calculated as the mean of the relevant items, with some items reverse-coded so that higher scores indicated greater expression of each trait. Scores were computed for participants with valid responses on at least half of the items for a given trait. All scales demonstrated sufficient reliability (Cronbach’s α .69 to .79).

#### Covariates

In the daily surveys, participants reported whether they had encountered a stressful event in any of the following domains of life that day: argument/disagreement, stressful event at work/school, stressful event at home, stressful event that happened to a friend, other stressful event. To control for stress exposure, a binary variable was created that indicated whether participants had encountered any stressor that day (0 = no stressful event, 1 = at least one stressful event) and the average amount of stress exposure throughout the study period (person-mean stress exposure). In the mail-out surveys, participants reported their age, sex, household income, whether they had taken any medication in the past 30 days (1 = yes, 0 = no), and their self-rated health (0 = worst possible health, 10 = best possible health). Additional covariates include the number of underage children living in the household and the age of the youngest child.

### Data Analysis

Two-level multilevel models with days (level 1) being nested within participants (level 2) were computed using the lme4 package^55^ in R Studio 2024.12.1 (running R 4.4.2;^56^). To disentangle between and within-person effects, time to oneself was person-mean centered prior to analysis. Person-mean time to oneself (aggregated over all 8 days) was entered on the day level to examine between-person effects. Continuous between-person (level 2) variables were centered on the sample mean. Models control for temporal changes in the outcome (study day; first day coded as 0) and include the random slope of daily time to oneself. Standardized regression estimates and pseudo-R2 were calculated for effect sizes. All analysis code can be accessed on the Open Science Framework (https://osf.io/an5ts/?view_only=57330a7aa49b40289462f2b3fd4033ef).

A first set of models predicted daily negative affect and daily wake-evening cortisol slopes by study day, daily time to oneself, person-mean time to oneself, and personality traits (main effects only model; H1). In a second step, the five interaction terms between daily time to oneself and personality traits were entered into the models (moderation model; H2). Sensitivity analyses tested whether findings remained consistent when including covariates.

Simulations using the simr package were conducted to calculate statistical power for this study^57^. Based on 318 participants providing an average of 7 out of 8 daily surveys and using an alpha-error rate of 5%, this study was able to detect small within-person effects of *r* = .10 with about 94% power and moderate cross-level interactions of *r* = .30 with about 90% power.

## Results

### Descriptive Results

Table 1 presents descriptive statistics and within- and between-person correlations of study variables. Participants reported positive solitude on 79% of days (*SD* = 0.25), on average. This was not significantly different between men (*M* = 0.79, *SD* = 0.24) and women (*M* = 0.80, *SD* = 0.26). Average negative affect was close to the lower end of the scale (*M* = 0.23, *SD* = 0.21). The average wake-evening cortisol slope was −0.61 (*SD* = 0.22).

Regarding bivariate between-person associations, older age (*r* = .18), having older children (*r* = .16), and experiencing fewer stressors (*r* = −.11) were significantly associated with more positive solitude. Taking medication (*r* = .14), worse self-rated health (*r* = −.23), and higher stressor exposure (*r* = .50) were associated with higher average levels of negative affect. Participants who were older (*r* = .16) and those who had older children (*r* = .18) showed less steep cortisol slopes.

All four daily assessed parameters showed significant variation on a day-to-day level (positive solitude: 75%; negative affect: 61%; cortisol slope: 73%; daily stressors: 84%). As expected, bivariate repeated measures correlations showed that days with no time to oneself were characterized by higher negative affect and less steep cortisol slopes. Bivariate within-person correlations showed that on days when participants reported positive solitude, they reported lower levels of negative affect (*r* = −.09) and steeper cortisol slopes (*r* = −.11). On days when negative affect was increased, participants showed less steep cortisol slopes (*r* = .09). On days, when participants reported a stressor, they reported higher levels of negative affect (*r* = .35) and less positive solitude (*r* = −.06).

### Main Results

#### H1: Positive Solitude as a Predictor of Negative Affect and Cortisol

**Figure 1.**
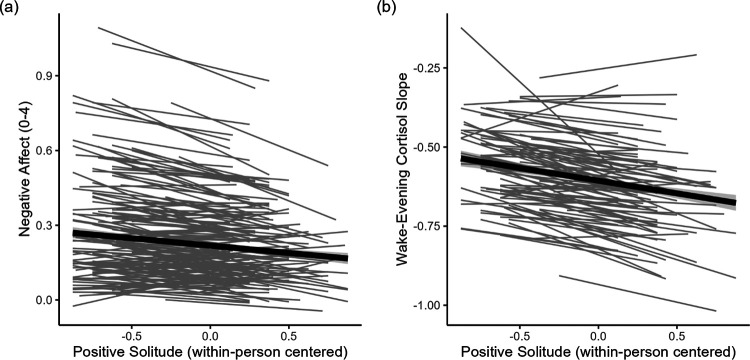
Associations of positive solitude with daily negative affect and salivary cortisol. *Note*. Parents showed a greater decrease in negative affect (panel a) and a greater decrease in salivary cortisol levels throughout the day (panel b) when they reported having had time to themselves.

Multi-level models predicting negative affect and salivary cortisol by positive solitude and personality, controlling for stressor exposure and study day can be found in Table 2. As hypothesized, parents were more likely to report lower levels of negative affect (*β* = −0.05, *SE* = 0.02, *p* = .005) and steeper wake-evening cortisol slopes (*β* = −0.10, *SE* = 0.03, *p* = .005) on days on which they experienced positive solitude (see Figure 1). Daily stressor exposure was associated with increased levels of negative affect (*β* = 0.27, *SE* = 0.02, *p* < .001) but not with cortisol slopes (*β* = 0.07, *SE* = 0.04, *p* = .061). Person-mean positive solitude and person-mean stressor exposure were not related to cortisol slopes, but participants with higher person-mean stressor exposure reported increased levels of negative affect, on average (*β* = 0.16, *SE* = 0.03, *p* < .001).

With respect to personality, higher levels of neuroticism (*β* = 0.21, *SE* = 0.03, *p* < .001) and openness (*β* = 0.09, *SE* = 0.04, *p* = .010) and lower levels of extraversion (*β* = −0.09, *SE* = 0.04, *p* = .016) were linked to higher average levels of negative affect. Parents with lower levels of conscientousness (*β* = 0.11, *SE* = 0.05, *p* = .023), lower levels of agreeableness (*β* = 0.14, *SE* = 0.05, *p* = .009), and higher levels of openness (*β* = −0.12, *SE* = 0.05, *p* = .021) showed steeper cortisol slopes, on average.

Explained variance in negative affect was 24.3% for fixed effects and 47.2% for fixed and random effects. Explained variance in wake-evening cortisol slopes was 5.4% for fixed effects and 36.9% for fixed and random effects.

#### H2: Personality as a Moderator of Associations of Positive Solitude with Daily Well-Being

**Figure 2.**
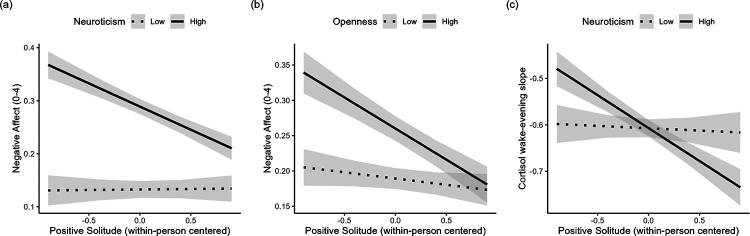
Within-person relationships between positive solitude, negative affect, and salivary cortisol moderated by personality *Note*. Individuals high in neuroticism and openness experienced decreased negative affect on days they had time to themselves, while this association was not significant for individuals low in neuroticism or openness. Additionally, those high in neuroticism showed steeper cortisol slopes on days with positive solitude, whereas no significant association was found for individuals low in neuroticism.

Next, interactions between positive solitude and personality in predicting negative affect and salivary cortisol were introduced into the multi-level models to test for moderating effects. The tables containing all parameters are available on the project’s OSF page (https://osf.io/an5ts/?view_only=57330a7aa49b40289462f2b3fd4033ef). The within-person association between positive solitude and negative affect was moderated by neuroticism (*β* = −0.05, *SE* = 0.02, *p* = .006) and openness (*β* = −0.04, *SE* = 0.02, *p* = .035). As can be seen in Figure 2, individuals high in neuroticism (*β* = −0.09, *SE* = 0.02, *p* < .001) and in openness (*β* = −0.09, *SE* = 0.02, *p* < .001) experienced a significant decrease in negative affect on days when they reported having had time to themselves. This was not true for individuals low in neuroticism (*β* = 0.00, *SE* = 0.02, *p* = .937) or openness (*β* = −0.02, *SE* = 0.02, *p* = .408). When all personality traits were entered into the same model, personality did not moderate associations between positive solitude and wake-evening cortisol slopes. However, in a model with neuroticism entered as a moderator, only, individuals high in neuroticism showed a stronger association between positive solitude and salivary cortisol (*β* = −0.04, *SE* = 0.01, *p* = .023). Specifically, individuals high in neuroticism experienced a decline in cortisol on days when they reported having had time to themselves (*β* = −0.14, *SE* = 0.04, *p* < .001), whereas for individuals low in neuroticism positive solitude was not significantly associated with cortisol (*β* = −0.01, *SE* = 0.04, *p* = .811).

#### Sensitivity and Follow-Up Analyses

The results remained consistent after controlling for age, sex, household income, the number of underage children in the household, and the age of the youngest child (for both outcomes), as well as self-rated health and medication use (for cortisol slope as outcome). Therefore, more parsimonious models without covariates are presented.

To explore potential time-ordered relationships, time-lagged effects from one day to the next were tested. Positive solitude on the previous day was not associated with next-day negative affect or cortisol slope after accounting for current-day positive solitude and both current and prior day stress exposure. Furthermore, follow-up models tested whether positive solitude would buffer the effect of daily stressors on well-being. The interaction between daily stressors and positive solitude on daily negative affect and cortisol slope was not significant.

Participants also reported the number of hours spent on leisure activities each day. The pattern of findings mirrors those observed for positive solitude: On days when participants spent more time on leisure activities than usual, they reported lower negative affect, holding stress exposure constant (*β* = −0.04, *SE* = 0.02, *p* = .030). Leisure time was also significantly associated with cortisol slope—days with more time spent on leisure were linked to a steeper cortisol decline (*β* = −0.08, *SE* = 0.04, *p* = .034).

Associations of positive solitude with negative affect and cortisol did not significantly differ by gender, age, the age of youngest child, or number of underage children in household. However, there was a significant three-way interaction between gender, neuroticism, and positive solitude on negative affect (*β* = −0.08, *SE* = 0.04, *p* = .034). Specifically, men high in neuroticism showed an even greater reduction in negative affect on days they had time to themselves, compared to men low in neuroticism.

## Discussion

As parents, the time cost to time to oneself is estimated to be about 4hrs per day for each younger child ≤ 3 years and 1.5 hrs per day for each child > 3 years^29^. Existing theories of social interaction suggest that social withdrawal can serve a restorative function, helping individuals recover and better prepare for future social and caregiving demands^12^. Thus, positive solitude could play an important part for the daily health and well-being of parents of underage children. Indeed, it was found that parents reported lower levels of negative affect and displayed steeper cortisol slopes (indicating better physiological stress recovery) on days on which they experienced positive solitude, as compared to days without positive solitude. These associations were moderated by parents’ personality traits, with individuals high in neuroticism and openness experiencing stronger positive correlates of positive solitude.

### Benefits of Positive Solitude for Parenting

Among the various restorative functions of positive solitude, previous literature has pointed out its role in emotion regulation^14^. In this study, parents reported lower levels of negative emotions—such as anger, fear, frustration, and sadness—on days when they had some time to themselves. Supporting this, a daily diary study found that individuals tend to seek solitude particularly when experiencing high-arousal negative emotions like irritation or anxiety^58^. Similarly, Bradshaw et al.^59^ found that even brief periods of solitude—as short as five minutes—led to mood improvements and reduced feelings of depletion. Solitude allows space to process difficult problems or feelings in private^60^ and has been shown to help down-regulate emotional arousal whether positive or negative^14^. This calming or “deactivation” effect may be especially important for parents of young children, who face a continuous stream of demands, from household responsibilities to emotional and physical caregiving^32^. Related research with middle-aged and older informal caregivers suggests that solitude can foster self-connection and enhance well-being—particularly when approached with a mindset of self-kindness^61^.

Days of positive solitude were also associated with steeper cortisol slopes, indicating a healthier diurnal cortisol rhythm. In contrast, a flattened cortisol slope (marked by less decline of cortisol levels throughout the day and increased evening levels) is often seen as a physiological indicator of chronic stress, burnout, or poor stress recovery^62,63^, suggesting that the body remains in a heightened state of arousal. People are more likely to seek solitude on days when their stress levels are elevated^43^. For parents, using moments of solitude for self-care—such as napping, listening to soothing music, or engaging in calming and restorative activities like painting or physical exercise—may offer important opportunities for psychological disengagement and emotional recovery from daily pressures^32^. Chronic parental stress has been linked to greater emotional exhaustion, increased risk for internalizing problems such as depression and anxiety, and reduced life satisfaction^27^. These stress-related effects can ripple outward, affecting relationship quality between partners^64^ and parenting, ultimately effecting the overall well-being of children^65^. For instance, studies with college students have found that insufficient time alone was associated with elevated levels of trait aggression, anger, and violent tendencies, as well as more aggressive behavior in experimental settings—such as inserting more pins into a voodoo doll^66^.

However, the potential downsides of spending too much time alone should not be overlooked^21^. Extended or frequent periods of solitude have been linked to lower daily well-being, increased negative affect, lower life satisfaction, and other adverse psychological outcomes^9,15,43^. Future research is needed to better understand where the tipping point lies—when beneficial solitude turns into social withdrawal or maladaptive disengagement^67^. It is also important to recognize that while positive solitude can restore energy, many parents experience time spent with their children as deeply fulfilling and restorative. In young mothers, personal time does not necessarily involve physical separation from the baby; instead, it can coincide with moments of rest and relaxation^32^. In a study of 186 parents of minor children, participants reported on their daily activities and rated how much they enjoyed each one. Interestingly, time spent on childcare was associated with greater positive affect than any other activity, including watching TV or cooking^68^.

### Personality Differences in Benefits of Positive Solitude

Associations of positive solitude with daily negative affect and cortisol slopes were moderated by neuroticism and openness. Specifically, only individuals high (but not those low) in neuroticism showed decreased negative affect and steeper cortisol slopes on days when they had time to themselves. Neuroticism was also associated with higher overall levels of negative affect throughout the study period. This is in line with previous research demonstrating that individuals high in neuroticism tend to be more irritable, anxious, impulsive, worrisome, tense, fearful, and high-strung—that is, they exhibit lower emotional stability^45^.

In the context of parenting, these emotional tendencies may amplify the stress associated with daily caregiving demands^42^. As such, parents high in neuroticism may benefit more from positive solitude to regulate affect, recover from emotional strain, and prevent overwhelm. Indeed, Ren et al.^47^ reported that individuals high in neuroticism perceived the emotion regulation function of solitude to be more important, as compared with individuals low in neuroticism. Moreover, individuals experiencing high-arousal negative emotions have been found to prefer solitude over social interaction; however, this association was not moderated by levels of neuroticism in younger adults^58^. The decrease in negative affect on days with positive solitude was greater for men than for women high in neuroticism. This aligns with prior research indicating that the link between feelings of time strains for oneself and life satisfaction was stronger for fathers than for mothers^24^.

Neuroticism has also been linked to heightened physiological stress reactivity and a greater reliance on passive and less effective coping strategies^69^. While findings on the relationship between neuroticism and HPA axis reactivity remain mixed and somewhat unclear, some evidence suggests that neuroticism is associated with overall higher cortisol output^70^. Positive solitude may therefore help parents high in neuroticism in downregulating physiological arousal, potentially leading to lower evening cortisol levels and a steeper diurnal decline.

Openness also moderated the relationship between positive solitude and negative affect, such that individuals with higher levels of openness experienced a greater reduction in negative affect on days when they had time to themselves. Openness to experience reflects a broad tendency toward curiosity, imagination, arts, and a preference for variety and seeking out new experiences^45^. People high in openness have been found to value solitude as a means for creativity and self-exploration^47^. Relatedly, autotelic personality—marked by curiosity, intrinsic motivation, the ability to concentrate efficiently, a desire for challenge, and the ability to transform boredom into engagement—has been associated with a greater likelihood of experiencing flow, including during solitary activities^71^. In the context of parenting, individuals high in openness may particularly benefit from positive solitude, as it offers an opportunity to engage in personally meaningful or imaginative activities such as journaling, creative writing, painting, listening to music, or reading fiction^8^.

The association between positive solitude and parents’ daily well-being was not moderated by conscientiousness, agreeableness, or introversion. This is somewhat unexpected, given that introverts are often characterized as individuals who recharge through solitude and derive energy from spending time alone^44^. Previous research on introversion within the Five Factor Model (as used in this study) has produced mixed results. Some studies have found that introverts show a stronger preference for solitude, engage more frequently in solitary activities, and derive greater enjoyment from them, while others have found no significant differences in the enjoyment of alone time between introverts and extraverts^44,71,72^. It may be important to differentiate between distinct facets of introversion when examining their associations with the benefits of positive solitude^44^. For example, social introversion—defined as a preference for low levels of social interaction and measured as one dimension of the STAR Introversion Scale^73^—has been found to predict a higher frequency of voluntary solitude episodes^43^. This contrasts with other facets of introversion as measured by the STAR Scale, such as thinking introversion (a tendency toward introspection, imagination, and deep thinking) and anxious introversion (characterized by discomfort or self-consciousness in social settings;^73^. Whether solitude is actively chosen for its positive qualities or pursued as a way to avoid social interaction has been shown to significantly shape how solitude is experienced^74^; see Rodriguez et al.^75^ for a positive reappraisal intervention of solitude. Therefore, distinguishing between anxious introversion and social introversion may be particularly important for future research on the psychological correlates of solitude.

### Strengths, Limitations, and Future Directions

This study offers several key strengths that contribute meaningfully to the literature on solitude and daily well-being. First, the sample was relatively large and demographically diverse, comprising 318 parents recruited through random-digit dialing. The sample included nearly equal numbers of mothers and fathers of underage children, with 19% identifying as non-White, thereby enhancing the generalizability of findings across gender and racial groups. Second, the use of an experience sampling design that incorporated both subjective reports and a physiological marker of stress—salivary cortisol—provides a richer, ecologically valid understanding of parents’ daily experiences. Saliva samples were collected in participants’ natural environments, capturing diurnal cortisol patterns alongside self-reported experiences of positive solitude and affect. Finally, the study contributes to a lifespan perspective on solitude. While most previous research has focused on solitude in childhood, adolescence, or late adulthood, relatively little is known about its role in midlife^67^. By examining solitude in a midlife parenting context, this study helps fill an important gap in understanding how solitude functions during a life stage marked by high social and caregiving demands.

Despite these contributions, several limitations should be acknowledged. One key limitation concerns the study’s cross-sectional and observational design, which precludes causal conclusions. Although the findings suggest that positive solitude is associated with better daily well-being, experimental studies are needed to establish whether positive solitude actively improves stress and affect. Future research could test this by instructing parents to engage in brief periods of positive solitude (e.g., 15–30 minutes of intentional time to themselves each day) and assessing changes in subjective well-being and physiological stress markers over time.

There are also limitations related to sampling. The study was conducted in the United States, and it remains unclear how the findings generalize to parents in other cultural contexts. Cultural norms surrounding solitude likely differ depending on dominant self-construals: in independent cultures, solitude may be more valued and accessible, yet social networks may be weaker, potentially increasing vulnerability to loneliness^76^. Conversely, collectivistic contexts may offer stronger social support and solitude might be viewed as meaningful opportunity for introspection, reflection, and self-cultivation^1^. Jiang et al.^77^ found that exposure to Chinese culture, whether through ethnicity or residence, was associated with better affective experiences during solitude. Supporting this, Japanese participants reported more favorable beliefs about being alone than their American counterparts, and these beliefs were associated with lower levels of loneliness in both cultural groups^1^. Future work should investigate how cultural values, including interdependent/dependent self-construals, shape the meanings and outcomes of solitude in daily life.

Measurement limitations should also be noted. Positive solitude was assessed using a single binary item asking whether participants had time to themselves on a given day (yes/no), which may oversimplify the complexity of positive solitude experiences. Although similar patterns emerged when using the number of hours spent on leisure activities as a predictor, future research should incorporate more refined measures that distinguish between different types and qualities of solitude. For instance, whether solitude involves technology use, physical separation from others, or internal focus may significantly influence whether solitude is experienced as restorative^78^. Moreover, this study did not assess what specific activities parents engaged in during their time to themselves. Positive solitude used for passive entertainment like watching Netflix or social media, recreational activities like going for a run, contemplative activities like planning or reflecting, or not doing any activity at all (activity-less solitude) may have differing effects on daily well-being^44^.

Another important yet overlooked factor is control over solitude. For many parents—especially those caring for young children—alone time is not only limited but unpredictable^32^. For instance, mothers may only find time for themselves during naps, the length of which is difficult to anticipate. This lack of control can lead to frustration when solitude is interrupted or insufficient for meaningful rest or engagement^32^. Finally, future research should also include other, related personality constructs such as dispositional autonomy (acting in accordance with one’s authentic interests and values) and sensory processing sensitivity (a heightened responsiveness to environmental and emotional stimuli;^43,46^.

### Conclusion

This study leveraged an experience sampling design—the gold standard for capturing in vivo experiences—and drew on a subsample of the MIDUS (Midlife in the United States) study, a nationally representative longitudinal panel focused on health, well-being, and aging in midlife and beyond. Findings underscore the potential psychological and physiological benefits of positive solitude: on days when parents experienced time to themselves, they reported reduced negative affect and showed steeper cortisol slopes, indicating better stress recovery. Notably, these associations were moderated by personality, with individuals high in neuroticism and openness experiencing the strongest benefits. Given the significant time demands of parenting young children, positive solitude may serve as a valuable and often overlooked resource for supporting emotional and physiological well-being in daily life.

## Figures and Tables

**Table 1:** Descriptive statistics and correlations among study variables (*N* = 318).

Variable	*M*	*SD*	1	2	3	4	5	6	7	8	9	10	11
1. Age	40.06	7.54											
2. Sex (1 = male)	0.45	0.50	.04										
3. Household income	97434.71	65183.49	.20**	.21**									
4. # children in household	2.02	1.15	−.15**	−.01	.01								
5. Age of youngest child	7.61	5.19	.64**	−.13*	−.02	−.39**							
6. Taking any medicine (1 = Yes)	0.32	0.47	.15**	−.11	−.00	−.10	.12*						
7. Self-rated health (0–10)	7.52	1.48	−.05	−.04	.10	−.00	−.10	−.15**					
8. Stressor exposure	0.47	0.28	−.05	−.10	−.01	.13*	−.08	.02	−.05		−.06*	.35**	.07
9. Time to oneself (0–1)	0.79	0.25	.18**	−.02	.00	−.03	.16**	−.06	.05	−.11*		−.09**	−.11*
10. Negative affect (0–4)	0.23	0.21	.03	−.07	−.02	.05	−.05	.14*	−.23**	.50**	−.09		.09*
11. Wake-evening cortisol slope	−0.61	0.22	.16*	−.04	.01	−.01	.18**	.06	−.09	−.05	.07	−.02	

Note. Means and SD are person-weighted averages for time to oneself, negative affect, and wake-evening cortisol slope. Between-person correlations are below and within-person correlations are above the diagonal.

* *p* < .05,

** *p* < .01.

**Table 2:** Multi-level models evaluating positive solitude and personality as a predictor of daily negative affect (n = 2299 observations of N = 318 individuals) and salivary cortisol (n = 788 observations of N = 255 individuals)

	Negative Affect			Wake-evening Cortisol Slope		
*Predictors*	*β*	*SE*	*p*	*β*	*SE*	*p*
(Intercept)	0.00	0.03	**<.001**	−0.00	0.05	**<.001**
Positive solitude	−0.05	0.02	**.005**	−0.10	0.03	**.005**
Person mean positive solitude	−0.03	0.03	.360	0.07	0.05	.132
Stressor exposure	0.27	0.02	**<.001**	0.07	0.04	.061
Person mean stressor exposure	0.16	0.03	**<.001**	−0.04	0.05	.372
Neuroticism	0.21	0.03	**<.001**	0.02	0.05	.739
Extraversion	−0.09	0.04	**.016**	−0.01	0.06	.885
Openness	0.09	0.04	**.010**	−0.12	0.05	**.021**
Conscientiousness	−0.06	0.03	.055	0.11	0.05	**.023**
Agreeableness	0.07	0.04	.058	0.14	0.05	**.009**
Day in Study (0 = first day)	−0.07	0.02	**<.001**	0.05	0.03	.120
*Random Effects*						
Level-1 residual variance	0.05			0.05		
Random intercept variance	0.02			0.02		
Random slope variance (positive solitude)	0.01			0.02		
Correlation random intercept & slope	−0.54			0.22		
Marginal R^2^ / Conditional R^2^	0.243 / 0.472			0.054 / 0.369		

*Note*. All continuous predictors were grand-mean centered. Positive solitude was within-person centered. Marginal and conditional R^2^ values represent the variance explained by fixed effects alone and by the full model, respectively.

## Data Availability

The data utilized in this study (MIDUS Refresher 1 Daily Diary) are publicly available through the Inter-university Consortium for Political and Social Research (ICPSR) at https://www.icpsr.umich.edu/web/ICPSR/studies/37083.

## Code availability

Analytic code is available on the OSF (https://osf.io/an5ts/?view_only=57330a7aa49b40289462f2b3fd4033ef).
